# Panhandle Medical Society

**Published:** 1891-03

**Authors:** 


Panhandle Medical Society Organized.—The physicians of the Panhandle district met at Vernon, Feb. 24, 1891, at 2 p.m. Meeting called to order by Dr. J. E. Dodson. The following officers were elected by ballot: Dr. J. E. Dodson, of Vernon, president; Dr. F. F. P. Keller, of Chillicothe, 1st vice-president; Dr.

*Published in this number of the JOURNAL.—ED.

W. M. Burger, of Claude, 2nd vice-president; Dr. J. F. Roberson, of Vernon, secretary; Dr. G. L. Van Duersen, of Vernon, treasurer. Adopted constitution and by-laws of North Texas Medical Association. Committee on Credentials elected Dr. G. W. Roberts, R. F. LeMond and H. H. Rhoades, all of Vernon. Paper read by Dr. F. F. P. Kellar: "The Differential Diagnosis of Diphtheria and Membranous Croup." It was highly appreciated, as was one read by Dr. G. W. Roberts on "Diagnosis and Treatment of Small-pox." Adjourned to meet at Quanah, May 15th, 1891. The Panhandle Medical Association embraces the territory between Wichita Falls and Texline.

